# Insecticide resistance in *Aedes aegypti*: An impact from human urbanization?

**DOI:** 10.1371/journal.pone.0218079

**Published:** 2019-06-24

**Authors:** Tri Baskoro Tunggul Satoto, Hary Satrisno, Lutfan Lazuardi, Ajib Diptyanusa, Purwaningsih, Rumbiwati, Kuswati

**Affiliations:** 1 Center for Tropical Medicine, Faculty of Medicine, Public Health and Nursing, Universitas Gadjah Mada, Indonesia; 2 Kapuas District Health Office, Central Kalimantan, Indonesia; 3 Department of Public Health, Faculty of Medicine, Public Health and Nursing, Universitas Gadjah Mada, Indonesia; 4 Department of Parasitology, Faculty of Medicine, Public Health and Nursing, Universitas Gadjah Mada, Indonesia; 5 Health Laboratory of Central Sulawesi, Central Sulawesi, Indonesia; Faculty of Science, Ain Shams University (ASU), EGYPT

## Abstract

In the city of Magelang, Indonesia, the distribution of Dengue Haemorhagic Fever (DHF) cases tend to be clustered, ever changing along with human urbanization from 2014 to 2017. Although DHF cases have been less reported in the city of Magelang for the past 5 years, vector control measures by using insecticide space spraying, particularly permethrin, have been continuously performed. Current study aimed to detect *kdr* mutations associated with pyrethroid resistance in *Ae*. *aegypti* and to study possible association between insecticide resistance and DHF case distribution related to human urbanization. The study was a cross sectional study conducted in 3 sub-districts in the city of Magelang, Central Java, Indonesia. Eggs of *Ae*. *aegypti* collected from 195 sample households were reared and were tested for resistance to pyrethroids by using PCR. Primers AaSCF1 and AaSCR4, and primers AaSCF7 and AaSCR7 were used in detecting presence of mutation in VGSC IIS6 and IIIS6 gene, respectively. Fragments of amplified DNA were sequenced and were analyzed. Spatio-temporal using Standard Deviational Ellipse (SDE) was performed to obtain mapping of DHF case distribution trends. The total number of DHF case was 380 cases, with the most cases (158) occurred in 2015 and the least cases (66) reported in 2017. DHF case distribution was grouped into several clusters. SDE calculation demonstrated movement of DHF case in the direction to principal arterial road, suggesting link to urbanization. Gene sequencing demonstrated VGSC IIS6 gene mutation (S989P and V1016G) in *Ae*. *aegypti* collected from study areas, indicating resistance to permethrin. VGSC IIIS6 gene mutation was not found. Current study concluded that multiple *kdr* mutations associated with resistance to pyrethroid was detected in *Ae*. *aegypti*, and that human urbanization may have a role in the development of such resistance.

## Introduction

Dengue is one of the fastest growing viral mosquito-borne diseases affecting tropical and subtropical regions in the world, including Indonesia [1]. The disease is caused by dengue virus principally transmitted by *Aedes aegypti* [2]. Roughly two-fifth of global population live in high-risk areas for dengue transmission [3], with an estimation of 3.9 billion people are at risk of infection with dengue viruses [4]. The number of dengue cases has been increasing over the last five years with recurring epidemics, particularly in Indonesia, Thailand, and Myanmar [5]. Recent report by the Ministry of Health of Republic of Indonesia showed a total of 129,650 dengue cases in 2015, with case fatality rate (CFR) of 0.97% [6]. This was generally due to increases in vector density, and human population size and mobilization [7].

Approximately 60% of Indonesian citizen reside in Java Island where dengue is a problem. The city of Magelang located in Central Java Province, Indonesia, belongs to highly endemic area for dengue hemorrhagic fever (DHF). Increasing number of DHF cases has been reported since the early 2000s in the city of Magelang, at which the peak occurred in 2010 [8]. However, the number of DHF cases significantly dropped in 2011, fluctuated during 2014 to 2017, and the case report remained low ever since. Such change in the disease pattern has also been reported to be influenced by human movement [9]. Dengue has been extensively spreading to many areas, with the tendency of spreading to urban areas [10], and urbanization has had an impact on the epidemiological pattern of dengue, especially in less developed countries. This mainly due to overcrowding, and poor-quality infrastructures, housing, and sanitation [11]. In such situation, by taking into consideration geographical aspects of disease phenomenon, DHF case dispersion trend may be sketched by using standard deviational ellipse (SDE) analysis in Geographic Information System (GIS) tool [12]. SDE analysis has also been used in disease surveillance and control [13].

Although a licensed dengue vaccine recently became available, protection is incomplete, and its use has been somewhat limited [14]. Hence, *Aedes* control using insecticides remained a key intervention for dengue prevention, including in the city of Magelang. Organophosphates, such as temephos and malathion, have been applied since 1970s, followed by the use of synthetic pyrethroids (permethrin, cypermethrin, deltamethrin) from 1980s as of today [15]. Routine and prolonged use of adulticide by thermal fog or ultra-low volume malathion, permethrin, or deltamethrin [16], as well as aerosolized insecticides [17] against *Ae*. *aegypti* may lead to resistance to these insecticides [18]. Resistance to malathion was reported in several districts in Indonesia [15], including in Magelang [19]. Although permethrin has been used in Magelang since 2014, its resistance status in *Ae*. *aegypti* is poorly studied. Possible mechanisms of pyrethroid resistance in *Ae*. *aegypti* involve alterations of enzyme activity (metabolic resistance) and target site mutations [20]. Mutations in voltage-gated sodium channel (VGSC) may inhibit action of pyrethroids, leading to knockdown resistance (*kdr*) [21]. Either individually or in combination, multiple *kdr* mutations in V1016G [22], S989P [23] and F1534C [24] has been associated with pyrethroid resistance in *Ae*. *aegypti*. The role of the environment including human urbanization in shaping insecticide resistance in mosquitoes has been demonstrated [25], yet the role of human urbanization is rarely studied in detail.

Current study aimed to detect *kdr* mutations causing pyrethroid resistance in *Ae*. *aegypti* mosquitoes as major vector of dengue virus causing DHF in Magelang, Central Java, Indonesia. The study also aimed to find potential associations between the occurrence of insecticide resistance and local DHF case distribution related to human urbanization by using SDE approach.

## Materials and methods

### Study design

The study was a cross sectional study, conducted from December 2017 to March 2018 in the city of Magelang (110˚12’30” - 110˚12’52” E and 7˚26’28” - 7˚30’9” S) in Central Java, Indonesia. The study was conducted in 4 villages: Rejowinangun Utara, Gelangan, Cacaban, and Magelang.

### Ethics statement

The permission to conduct the study was approved by the Ethics Committee of Faculty of Medicine, Public Health and Nursing, Universitas Gadjah Mada (Reference No. KE/FK/1293/EC/2017). The Ethics Committee waived the requirement for informed consent and data were fully anonymized before analysis. Map of the city of Magelang was reproduced with permission from Regional Development Planning Agency.

### Sampling methodology

Data regarding local population and number of DHF cases from 2014 through 2017 were obtained from local health office. Incidence rate (IR) per 100,000 population was calculated afterwards.

Given total number of houses in all 3 sub-districts in the city of Magelang of 35,678 and House Index (HI) of >5%, generated sample size was 177 houses [26]. We estimated 10% of loss to follow-up, hence final sample size was 195 houses. Sampling was performed by using purposive sampling method from houses with reported DHF case in year 2017 and 2016. We selected the house alternatively every 100 m from the index case [27]. Ovitraps were installed in the selected houses. Ovitraps positive for *Ae*. *aegypti* eggs were calculated as percentage as Ovitrap Index (OI). Eggs of *Ae*. *aegypti* collected from 195 sample households were reared for *kdr* mutation analysis.

### *Kdr* mutation analysis

Reared mosquitoes were tested for resistance to pyrethroids by using PCR in the Laboratory of Parasitology, Faculty of Medicine, Public Health and Nursing, Universitas Gadjah Mada. Pooled mosquito samples consisting of 10 mosquitoes were lightly dried on a paper towel and were placed in a 1.5-mL PCR reaction tube. The sample was homogenized in a 40 mL of mixed solution of extraction solution and 10 mL of tissue-preparation solution (REDExtract-N-Amp Tissue PCR Kit; Sigma, St. Louis, MO) for DNA extraction. The solution was heated at 95°C for 3 minutes and was then neutralized. A total of 4 pooled mosquito tubes were tested for reared mosquitoes from each area.

The PCR was performed under the following conditions: (1) initial denaturation at 94°C for 3 minutes; (2) further run at 94°C for 15 seconds, 55°C for 30 seconds, and 72°C for 30 seconds for 35 cycles and; (3) final elongation step at 72°C for 10 minutes. The amplified fragments of the expected size were purified with ExoSAP-IT (USB Corporation, Cleveland, OH) at 37°C for 30 minutes and at 80°C for 15 minutes [28]. A total volume of 10 mL of PCR mixture contained 4 mL of REDExtract-N-Amp ReadyMix (Sigma), 0.5 mM of each primer, and 1 mL of DNA template.

Resistance to pyrethroids was demonstrated from presence of VGSC IIS6 and VGSC IIIS6 gene mutations of *Ae*. *aegypti* in gene sequencing analysis [29]. Current study used primers AaSCF1 (AGACAATGTGGATCGCTTCC) and AaSCR4 (GGACGCAATCTGGCTTGTTA) in detecting presence of mutation in VGSC IIS6 gene and used primers AaSCF7 (GAGAATCGCCGATGAACTT) and AaSCR7 (GACGACGAAATCGAACAGGT) in detecting presence of mutation in VGSC IIIS6 gene [30]. Fragments of amplified DNA were sequenced and were analyzed using MEGA 7.0.18 [31] and BioEdit ver. 7.2.6 (California, USA).

### Standard deviational ellipse analysis

Spatio-temporal approach was implemented in order to obtain mapping of DHF case distribution. Collected waypoint data regarding DHF cases were analyzed using the software ArcGIS ver. 10.4.1.5686 (Esri, New York) with SDE calculation to acquire mapping of DHF case distribution trend from 2014 through 2017. Urban densities over the years were also provided in the maps.

## Results

**Fig 1:**
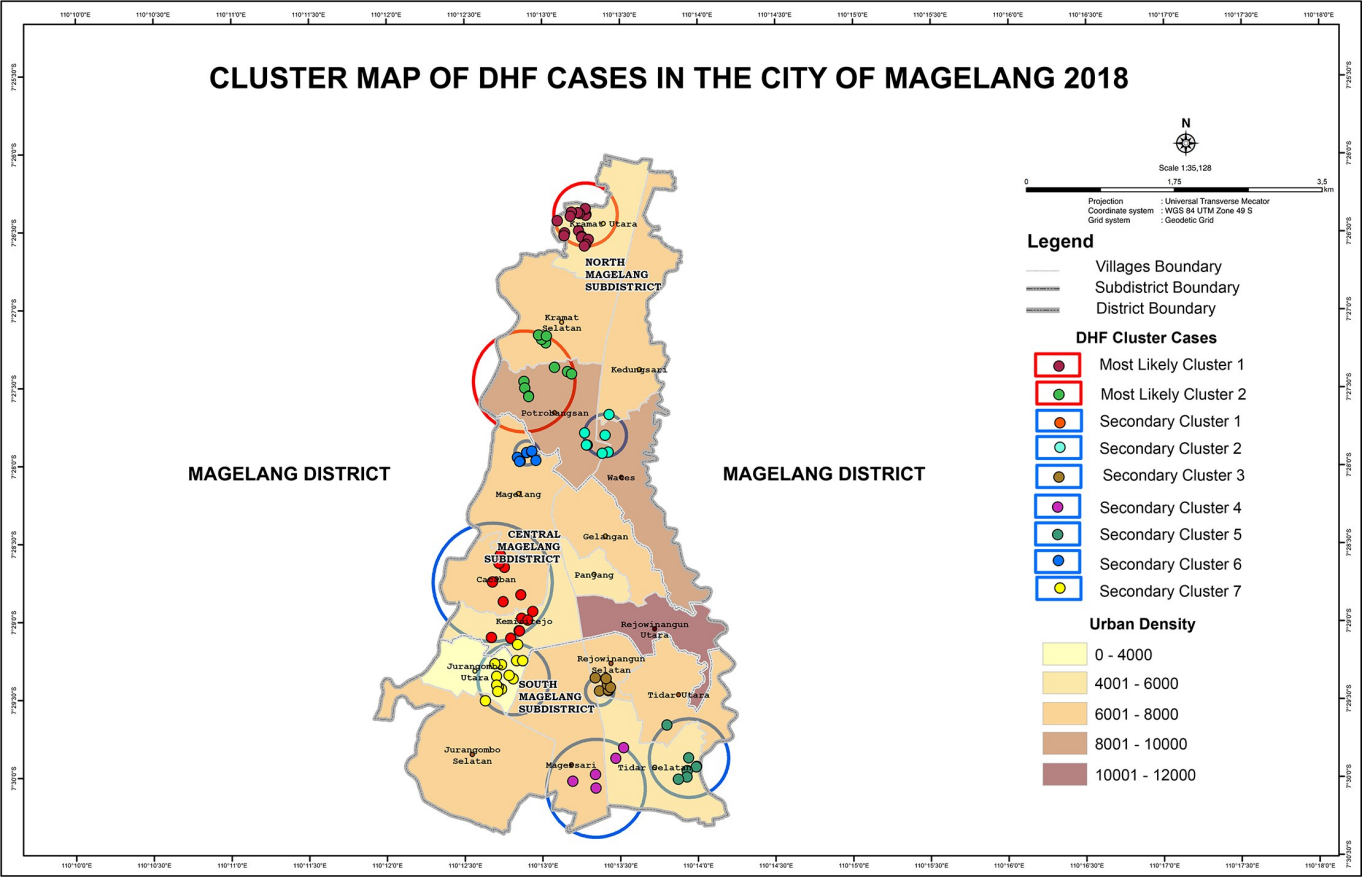
Cluster map of DHF in Magelang City year 2018.

In total, the number of reported DHF cases in study areas from 2014 through 2017 was 380 cases: 69 cases reported in 2014, 158 cases reported in 2015, 87 cases reported in 2016, and 66 cases reported in 2017. The IR per 100,000 population was observed highest in 2015 (Table 1). Two death cases were reported in 2015 and 4 death cases were reported in 2016. Table 1 also demonstrated population density along the years from 2014 through 2017. Population increase in general was subtle, however, the trend was observed highest in Central Magelang sub-district, showing tendency of human urbanization into city center. The distribution of DHF cases demonstrated a clustered pattern further divided into primary and secondary clusters (Fig 1).

**Table 1:** Population and incidence rate (IR) of DHF according to sub-districts and villages in the city of Magelang from 2014 through 2017.

Sub-districts and villages		2014			2015			2016			2017		
		DHF	Population	IR	DHF	Population	IR	DHF	Population	IR	DHF	Population	IR
*North Magelang*													
	Wates	2	7,903	25.31	12	7,927	151.38	4	8,195	48.81	6	8,219	73
	Potrobangsan	4	7,970	50.19	3	8,059	37.23	8	8,052	99.35	6	8,077	74.29
	Kedungsari	2	7,175	27.87	14	7,194	194.61	11	6,995	157.25	5	7,015	71.28
	Kramat Utara	11	5,716	192.44	16	5,769	277.34	1	5,727	17.46	2	5,743	34.83
	Kramat Selatan	1	7,463	13.4	7	7,576	92.4	8	7,370	108.55	5	7,391	67.65
*Central Magelang*													
	Rejowinangun Utara	0	10,566	0	4	10,599	37.74	1	10,653	9.39	4	10,683	37.44
	Kemirirejo	3	4,993	60.08	14	4,975	281.41	9	5,253	171.33	2	5,268	37.97
	Cacaban	4	7,768	51.49	10	7,802	128.17	3	7,752	38.7	0	7,773	0
	Magelang	9	6,963	129.25	13	6,932	187.54	4	7,086	56.45	3	7,105	42.22
	Panjang	2	5,722	34.95	3	5,711	52.53	0	5,895	0	3	5,912	50.74
	Gelangan	2	7,344	27.23	9	7,478	120.35	5	7,383	67.72	6	7,403	81.05
*South Magelang*													
	Magersari	2	7,834	25.53	3	7,894	38	7	7,859	89.07	3	7,882	38.06
	Rejowinangun Selatan	6	7,767	77.25	13	7,815	166.35	3	7,965	37.66	6	7,987	75.12
	Jurangombo Utara	5	3,848	129.94	9	3,908	230.3	5	3,894	128.4	1	3,903	25.62
	Jurangombo Selatan	7	7,851	89.16	10	7,942	125.91	6	7,698	77.94	9	7,718	116.61
	Tidar Utara	6	7,672	78.21	10	7,769	128.72	7	7,736	90.49	3	7,759	38.66
	Tidar Selatan	3	5,569	53.87	8	5,580	143.37	5	5,439	91.93	2	5,455	36.66
**Total**		**69**	**120,124**	**57.44**	**158**	**120,930**	**130.65**	**87**	**120,952**	**69.95**	**66**	**121,293**	**54.41**

IR: incidence rate (per 100,000 population)

**Fig 2:**
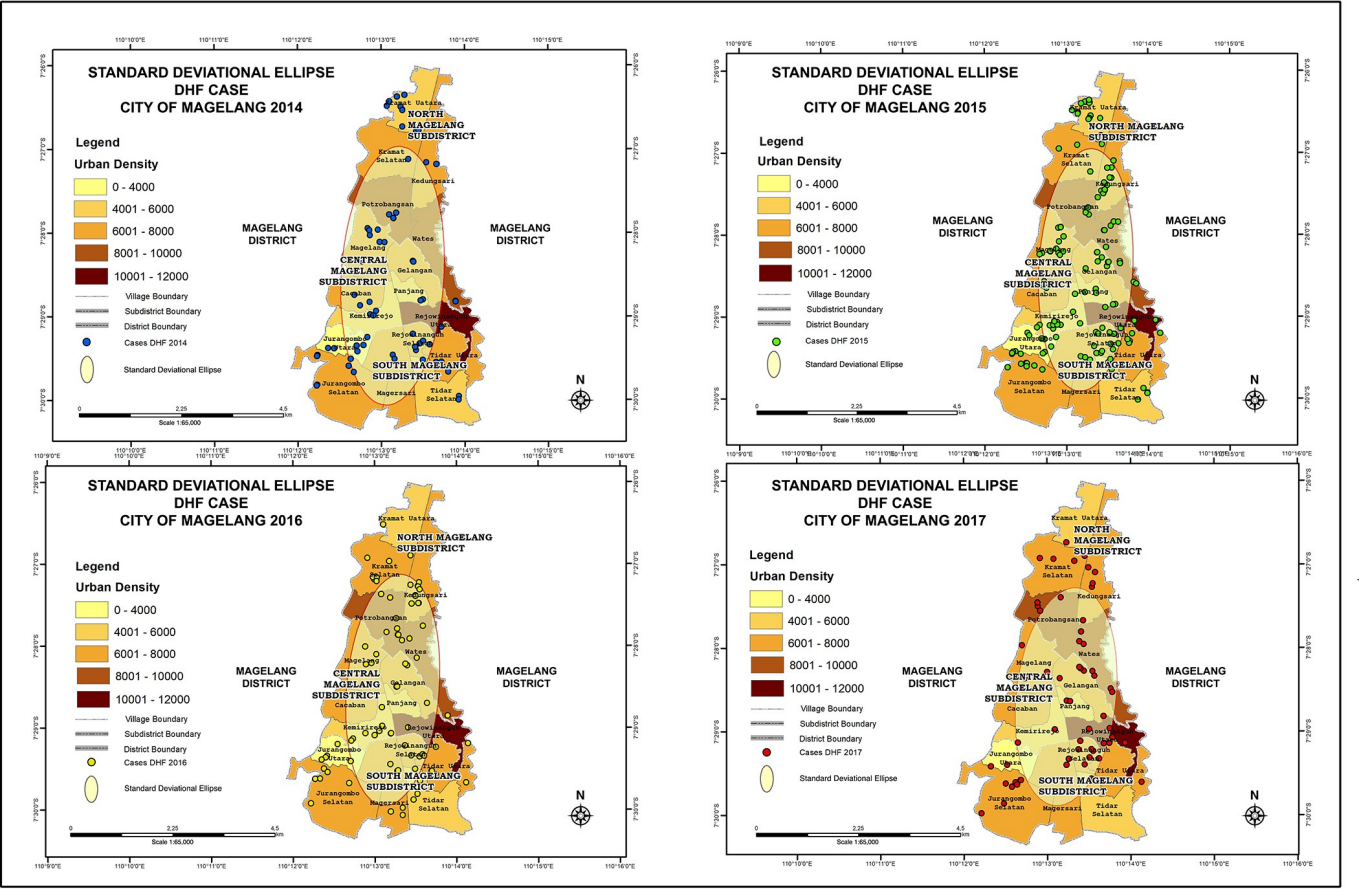
Standard deviational ellipse analysis of DHF cases in Magelang City year 2014 through 2017. The cases showed movement to the direction of main arterial road.

In 2014, the clusters were focused in North and South Magelang, while in 2015 the pattern slightly changed into concentrated clusters in Central and South Magelang. The number of DHF cases was significantly higher compared to other studied years. Similar distribution pattern to 2015 was observed in 2016 and 2017. Yearly DHF case distribution is depicted in Fig 2. There were no seasonal variations observed from the number of reported DHF cases in each month, each year. Standard Deviational Ellipse calculation demonstrated movement of DHF case in the city of Magelang 3.8^0^ Northeast, similar direction to principal arterial road heading to other neighboring provinces (Fig 2).

**Fig 3:**
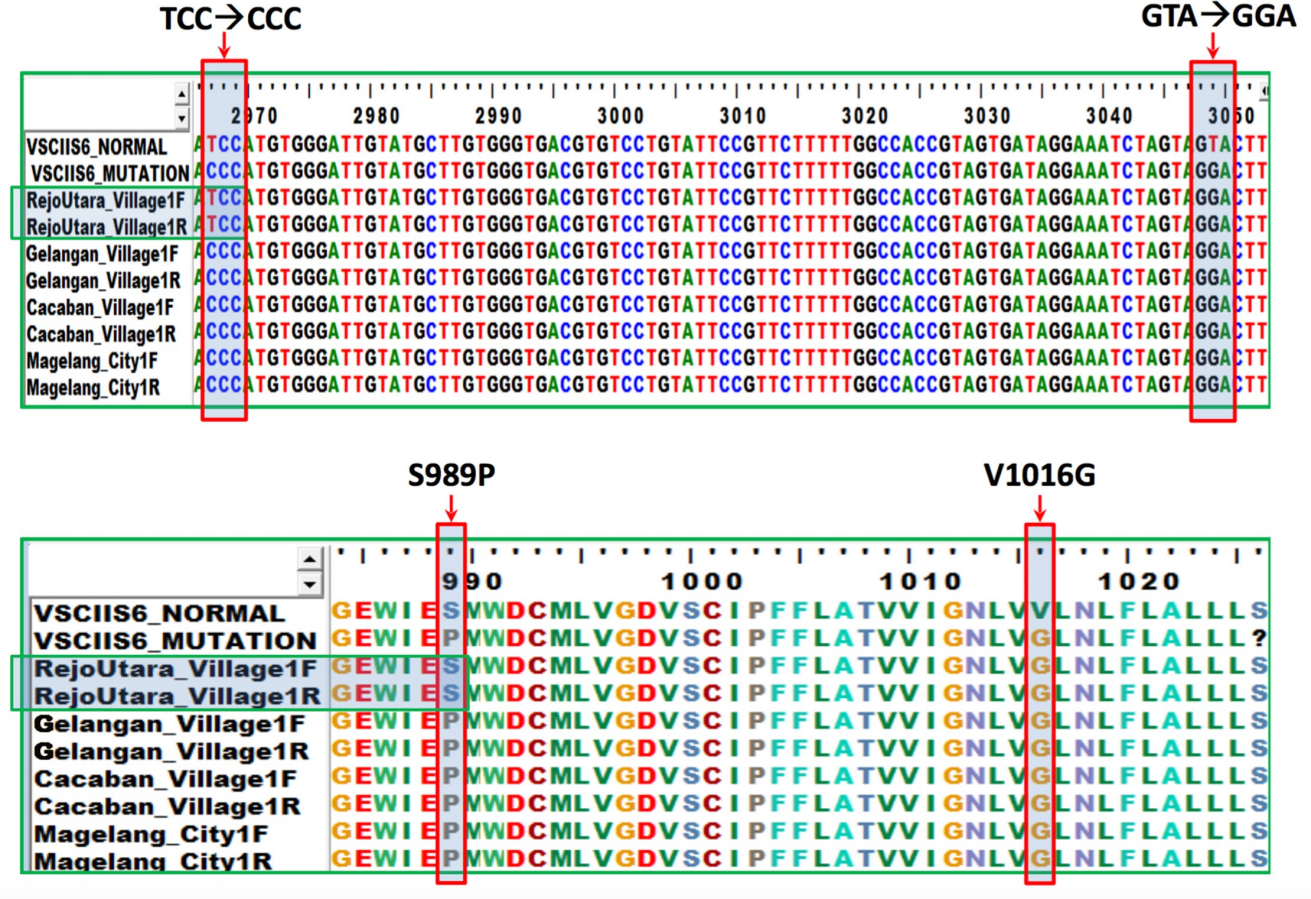
VGSC IIS6 gene mutation. VGSC IIS6 gene mutation from serine (TCC) to proline (CCC), and from valine (GTA) to glycine (GGA) at target site S989P and V1016G observed in samples obtained from Gelangan, Cacaban, and Magelang.

**Fig 4:**
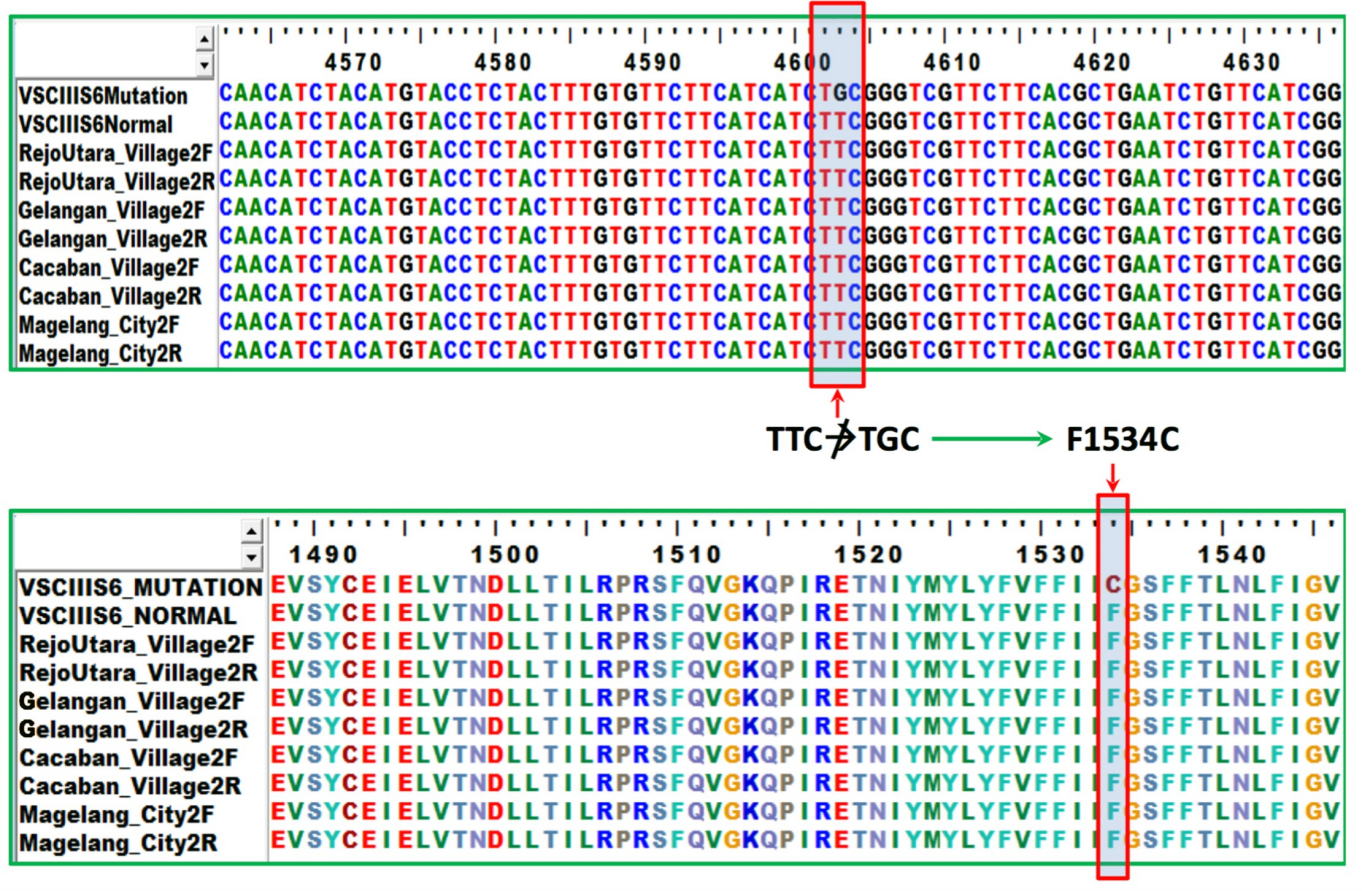
VGSC IIIS6 gene mutation. No observed mutation of VGSC IIIS6 gene, as phenylalanine (TTC) to cysteine (TGC) mutation did not occur at target site F1534C.

Installation of ovitraps was performed in 195 houses in 3 sub-districts in the city of Magelang, represented by 4 villages: Rejowinangun Utara, Gelangan, Cacaban, and Magelang. Among 195 installed ovitraps, 119 ovitraps showed positive results (OI of 61.03%). Collected *Ae*. *aegypti* eggs were reared and pools of 10 mosquitoes were tested for *kdr* mutations suggesting pyrethroid resistance. Sequencing analysis results were aligned with GenBank AB914689 and AB914690 for VGSC IIS6 gene mutation and aligned with GenBank AB914687 and AB914688 for VGSC IIIS6 mutation. Samples obtained from Gelangan, Cacaban, and Magelang demonstrated VGSC IIS6 gene mutation from serine (TCC) to proline (CCC), and from valine (GTA) to glycine (GGA) at target site S989P and V1016G as shown in Fig 3. Samples from Rejowinangun Utara only showed mutation from valine (GTA) to glycine (GGA) at target site V1016G. On the other hand, mutation of VGSC IIIS6 gene was not observed, as phenylalanine (TTC) to cysteine (TGC) mutation did not occur at target site F1534C (Fig 4). All samples obtained from Gelangan, Cacaban, and Magelang showed homozygous sequencing chromatograms. Samples collected from Rejowinangun Utara showed heterozygosity.

## Discussion

The city of Magelang, located in the middle of Magelang Regency, is a city in Central Java that is routinely affected by DHF. The city contributes 1.67% of Magelang Regency with 18.12 km^2^ of areas. The city is a fertile agricultural area and one of the most densely populated area in Central Java, with population density of 6,693/km^2^. As of 2017, population in the city of Magelang was 121,293 people, distributed in 3 sub-districts: North (36,445), Central (44,144), and South Magelang (40,704). Current study results showed the highest number of reported DHF case in 2015, along with the highest IR per 100,000 population. In the city of Magelang, permethrin was newly introduced in 2014 after prolonged use of malathion in space spraying. In Magelang, mosquitoes have been known to be resistant to malathion, as demonstrated by mosquito mortality of <50% throughout the city [19]. Since insecticide resistance to malathion was increased drastically in 2013 [MOH of Republic of Indonesia, 2017, unpublished], local government started using permethrin in insecticide space spraying in 2014 to reduce *Ae*. *aegypti* population as main vectors of DHF. The number of reported DHF case was decreased significantly after introduction of permethrin. However, the number of DHF case showed dramatic increase in 2015. This may be due to several reasons: increased mosquito population, human movement, increasing trades, or insecticide resistance [7, 32]. Furthermore, DHF incidence decreased after year 2015 most likely because local government was concerned by drastic increase of DHF incidence that programs involving source reduction and environmental management were intensified in the community ever since [8].

The DHF case distribution demonstrated that the pattern tended to change, initially focused in northern parts in 2014 and the clusters slowly moved to southern part of the city in 2017. Standard Deviational Ellipse results in this study showed that the movement of DHF case leaned towards the direction on main arterial road over the years. Such results proved that distribution of DHF case moved along the more urban area following human urbanization. However, we did not perform confidence level analysis for the SDE in year 2014 and 2017, therefore we could not demonstrate statistical difference between the SDE results. Formulas for calculation of tabulating confidence intervals in SDE analysis have been proposed [12]. In this study, albeit subtle increase in population, Central Magelang sub-district showed highest population increase, showing tendency of human urbanization into city center along with higher incidence of DHF in 2015. Another spatio-temporal study in Thailand also showed DHF diffusion pattern to urban fringe [33]. Human urbanization to the center of the city might also be associated with human activities including trades. Certain trades, such as tires, have been stated to correlate with increasing DHF incidence, as those trading goods acted as breeding places for mosquitoes [34]. Other than the rise of urban civilization and trades, peridomestication of *Ae*. *aegypti* and *Ae*. *albopictus* also resulted in DHF pattern change into urban areas [35].

Aside from aforementioned factors, one potential contributing factor of increase in DHF incidence is resistance to insecticides. Current study demonstrated multiple *kdr* mutations suggesting pyrethroid resistance, shown by the presence of VGSC IIS6 gene mutation at target sites S989P and V1016G. Such results indicated decreased mosquito target site sensitivity against pyrethroid, particularly permethrin. First permethrin resistance in Indonesia was reported to occur in Semarang in 2003 [36]. Many countries in Southeast Asia have reported permethrin resistance in *Ae*. *aegypti* as well [37–39]. These studies also found serine to proline mutation (S989P), either with valine to glycine mutation (V1016G) or phenylalanine to cysteine (F1534C). Conversely, less gene mutation was found in mosquitoes from Rejowinangun Utara village, as the mosquito only showed mutation from valine (GTA) to glycine (GGA) at target site V1016G. This may explain constantly lower number of reported DHF cases each year from the village, showing lesser degree of resistance. *Kdr* mutations in V1016G, S989P and F1534C, either individually or in combination, has been linked resistance phenotypes of *Ae*. *aegypti* against pyrethroids [40]. This is supported by other studies [40–43]. However, equating only *kdr* mutations with resistance is probably misleading [44]. Bioassay testing for permethrin was not performed in this study, assuming that high resistance to malathion demonstrated by previous study [19] might have caused tolerance to pyrethroids [45]. Current study showed multiple *kdr* mutations, indicating that use of pyrethroids may not be efficacious in study area [44]. One of the most commonly used vector control programs to reduce *Ae*. *aegypti* population in Indonesia is insecticide spraying. Considering this situation, quantification of resistance and genetic markers of insecticide resistance is necessary for more timely information on area-specific resistance levels, hence better operational decision-making [46].

Interestingly, previous bioassay study in the same areas in Magelang by using 0.8% malathion impregnated paper showed resistance in *Ae*. *aegypti* mosquitoes [19]. Insecticide resistance in mosquitoes can occur simultaneously against several groups of insecticides [47, 48]. Susceptibility status of mosquitoes is associated with genetic, biological, and operational factors [49, 50]. Resistance to permethrin and malathion in Magelang may be due to routine use of malathion and permethrin in space spraying without knowing the susceptibility status [51]. Residents in Magelang also tend to use household sprays containing pyrethroids, as these are the cheapest and most available choice as adulticides available in the area. The use of insecticide will inevitably form resistance in mosquito populations. The presence of permethrin resistance in the city of Magelang has been demonstrated only after several years of permethrin use in the localities. Although insecticide resistance usually develops after 2 to 20 years of use, it may develop faster in prolonged, routine use of insecticide [52, 53].

Growth of towns and cities, or urbanization, may provide new mosquito breeding places, as this is associated with increasing human waste, poor sanitation, and inferior housing, all of which possibly affect the distribution of mosquito vectors [54, 55]. Current study showed increasing population size and human urbanization from less-urban areas to urban areas in almost the same period as the occurrence of insecticide resistance. This population may have practiced the use of insecticide spraying, as they usually do in agricultural field in the cities, which may lead to development of insecticide resistance in mosquitoes [56]. Small-scale urban agriculture and uncontrolled use of pesticides have been reported to cause insecticide resistance as well [57]. Several studies also demonstrated that urban pollutants potentially play a role in the development of insecticide resistance by altering mosquito detoxification system that leads to enhanced tolerance to insecticides [58, 59]. Pursuing the study regarding impact of urbanization on insecticide resistance in mosquito vectors will ensure particular attention to highly vulnerable urban environments, where the risk of dengue transmission is highest.

## Conclusions

The use of permethrin in the city of Magelang, Central Java, Indonesia has been routinely applied in vector control since 2014 after decades of malathion use. Current study demonstrated multiple *kdr* mutations linked with pyrethroid resistance in *Ae*. *aegypti* mosquitoes collected from the city of Magelang, as evidenced by the presence of VGSC IIS6 gene mutation (S989P and V1016G). In the city of Magelang, development of insecticide resistance may be potentially be related to slight increase in population size and human urbanization.
